# Reactive Transport Modeling of the Enhancement of Density-Driven CO_2_ Convective Mixing in Carbonate Aquifers and its Potential Implication on Geological Carbon Sequestration

**DOI:** 10.1038/srep24768

**Published:** 2016-04-20

**Authors:** Akand Islam, Alexander Y. Sun, Changbing Yang

**Affiliations:** 1 Bureau of Economic Geology, The University of Texas at Austin, TX, USA

## Abstract

We study the convection and mixing of CO_2_ in a brine aquifer, where the spread of dissolved CO_2_ is enhanced because of geochemical reactions with the host formations (calcite and dolomite), in addition to the extensively studied, buoyancy-driven mixing. The nonlinear convection is investigated under the assumptions of instantaneous chemical equilibrium, and that the dissipation of carbonate rocks solely depends on flow and transport and chemical speciation depends only on the equilibrium thermodynamics of the chemical system. The extent of convection is quantified in term of the CO_2_ saturation volume of the storage formation. Our results suggest that the density increase of resident species causes significant enhancement in CO_2_ dissolution, although no significant porosity and permeability alterations are observed. Early saturation of the reservoir can have negative impact on CO_2_ sequestration.

It has been widely recognized that a significant reduction of CO_2_ emissions is necessary to maintain atmospheric greenhouse gas concentrations at around 450 ppm CO_2_ equivalent, thus limiting the effect of anthropogenic climate change^1^. Reduction targets suggest a 30% reduction of 1990-level by 2020 and even up to 80% by 2050 (commission of the European communities, 2007). The feasible technology to achieving targets is Carbon Capture and Storage (CCS). In general, injection of CO_2_ under supercritical conditions into geological formations such as saline aquifers has been proposed to sequester CO_2_ over a long period of time^2^,^3^. Deep saline aquifers are estimated to have the greatest capacity for CCS, 10^3^–10^4^ gigatons^4^.

The injection of a reactive substance such as CO_2_ into the saline formation results in chemical disequilibration and initiation of various chemical reactions. It is important to understand the direction, rate, and magnitude of such reactions, both in terms of their impact upon the host rocks’ ability to contain the injected CO_2_ and in the long-term stability of CO_2_ containment^5^. Structural, residual, solubility, and mineralogical trapping are the dominant mechanisms by which the injected CO_2_ is contained^6^,^7^,^8^,^9^. Among these, mineralogical trapping is the only mechanism that permanently sequesters the CO_2_ while others are regarded as storage processes^10^. This permanent sequestration ensures long term stability.

After injection, mass transfer at the interface between CO_2_ and brine occurs by molecular diffusion of CO_2_ into brine. A 2–3% density increase of brine-CO_2_ solution leads to gravitational instability. Under favorable conditions (greater than critical Rayleigh number, *Ra*), natural convection enhances dissolution further^11^. An increase in dissolved minerals further increases the density of the formation water which, in turn, enhances natural convection. In addition, precipitation and dissolution of minerals may lead to an alteration in porosity and permeability of the host formation. Consequently, convective mixing and, therefore, CO_2_ solubility trapping are influenced.

Several studies including a few laboratory tests have been performed regarding convective mixing from perturbed saturated boundary^11^,^12^,^13^,^14^,^15^,^16^,^17^,^18^,^19^. However, the geochemical effect to this end is modeled to a lesser extent. Ennis-King and Paterson^20^ investigated analytical and numerical methods and observed that the overall dissolution rate depends on the balance between the effects of permeability alterations and the consumption of dissolved CO_2_. They showed that the influence of ion concentrations (e.g., Ca^+2^, Mg^+2^) on the fluid density alters the plume structure and favors faster dissolved plume development. Ghesmat *et al*.^21^ used linear stability theory and direct numerical simulation to show that geochemical reactions stabilize the unstable diffusion boundary layer because of the consumption of dissolved CO_2_. Their results implied that more CO_2_ can be trapped through mineral interactions. Zhang *et al*. classified dissolution-diffusion-convection process into four stages: (1) dissolution-dominated period; (2) diffusion-dominated period; (3) early convection-dominated period; and (4) late convection-dominated period^22^. Islam *et al*.^23^ presented simulation results of convective mixing with reactions adding heterogeneity and geothermal effects. They concluded that at a fixed Damkohler number (*Da*), reaction orders make substantial difference of mixing over longer period of times. Geothermal gradient exhibited negligible impact. Ward *et al*.^24^ studied reactive flow in the limit of high *Ra* in which the domain considered was deep, shallow or of intermediate depth, and for which the *Da* was respectively large, small or of order unity. For large *Da* the rapid reaction rate limits the plume depth and the boundary layer restricts the rate of solute transfer to the bulk volume, whereas for small *Da* the average solute transfer rate is ultimately limited by the domain depth and the convection is correspondingly weaker. All these previous researches were very generalized, in terms of *Ra* and *Da*.

Fu *et al*.^25^ presented formation of rock-dissolution patterns that arose from a series of calcite dissolution reactions during convection. They used high-resolution simulations to examine the interplay between the density-driven hydrodynamic instability and the rock dissolution reactions and analyzed the impact on the macroscopic mass exchange rate. Their conclusion was the geochemical reactions terminate significantly earlier than the time when convective mixing stops. However, it should be clearly noted that geochemical effects may not always accelerate advection. A precipitation reaction such as that between the acidic brine and a rock formation rich in calcium feldspar promotes the deposition of solid calcite and kaolinite, removing CO_2_ from the liquid phase. Such a reaction may actually attenuate convection motion^26^. Dai and co-authors^27^ developed an integrated Monte Carlo method for simulating CO_2_ and brine leakage from carbon sequestration and subsequent geochemical interactions in shallow aquifers. Their results showed shallow groundwater resources may degrade locally by reduced pH and increased total dissolved solids. Ennis-King and Paterson^20^ and Fu *et al*.^25^ showed results of porosity and permeability changes from reactions and subsequent impacts on convection mechanism. However, in this work we show, by performing reactive transport modeling with speciation of a series of calcite and dolomite dissolution reactions, that dissolution enhancement causes local density increase and effects from porosity and permeability change are almost negligible even when the reservoir reaches near 100% dissolved CO_2_ saturation.

## Results and Discussions

**Figure 1:**
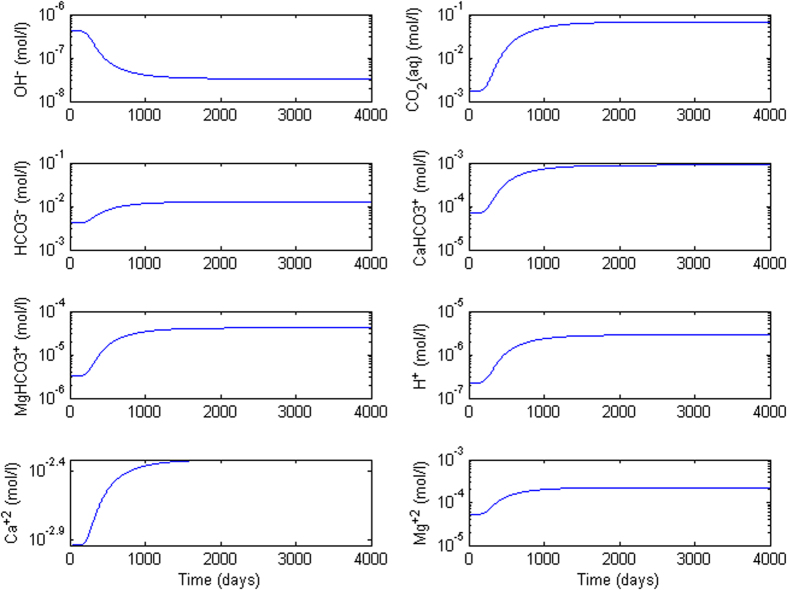
Equilibrium concentrations after breakthrough.

We first have conducted sanity test of chemical speciation. CO_2_ leak problem is solved considering chemical reactions of Eqs 3, 4, 5, 6, 7, 8 by following the semianalytical approach in Yang *et al*.^28^. It is noteworthy that coupled reactive transport calculations strongly depend on accuracy of geochemical database. Different geochemical databases and uncertainty of thermodynamics data can severely affect the results. For the transport part analytical solution for the scenario with leaky well was used. Figure 1 shows that CO_2_ stream in host carbonates after equilibrium dissolution can be increased by more than 1.5 folds. In our simulation the maximum equilibrium dissolved CO_2_ concentration of 0.97 mol/l is used in saturated CO_2_-brine interface at the top. In order to quantify saturation area of the aquifer, average concentration formulation, 
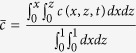
, is used. The direct numerical simulations have been carried out for *Ra* numbers of 1000 and 10,000, where the associated mean permeability values are 10 and 100 mD, respectively.

**Figure 2:**
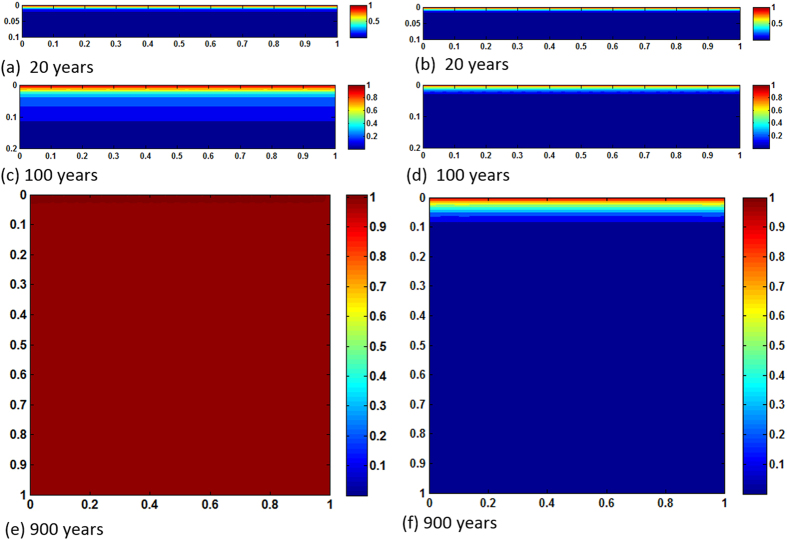
Concentration maps of *Ra* = 1000, homogeneous case; (**a,c,e** and **b,d,f**) show results of with and without reactions, respectively.

**Figure 3:**
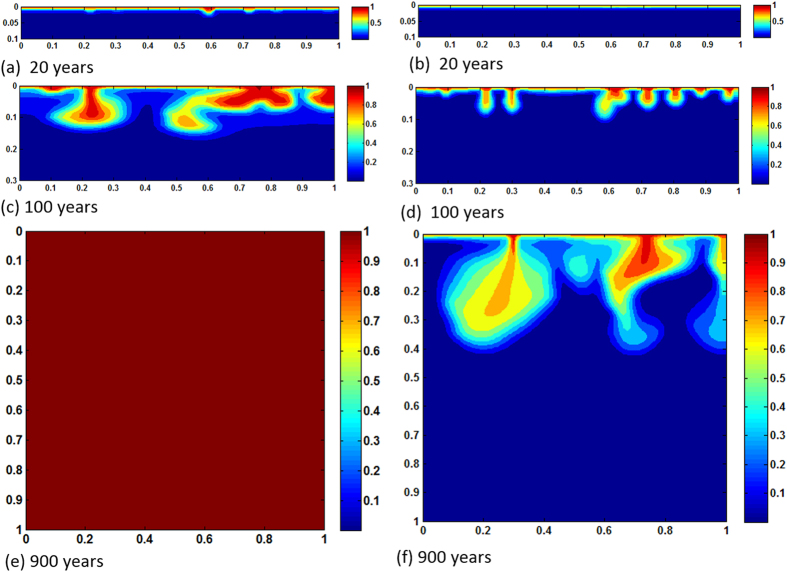
Concentration maps of *Ra* = 1000, heterogeneous case; (**a,c,e** and **b,d,f**) show results of with and without reactions, respectively.

**Figure 4:**
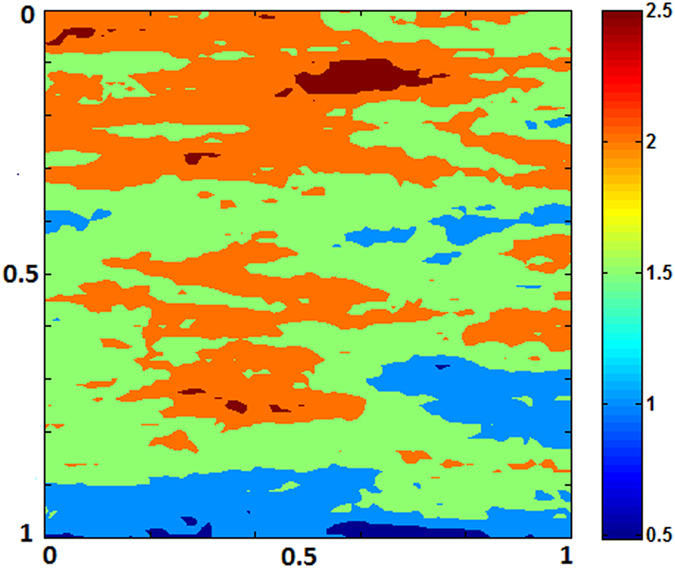
log_10_*k* distributions of heterogeneous reservoir.

**Figure 5:**
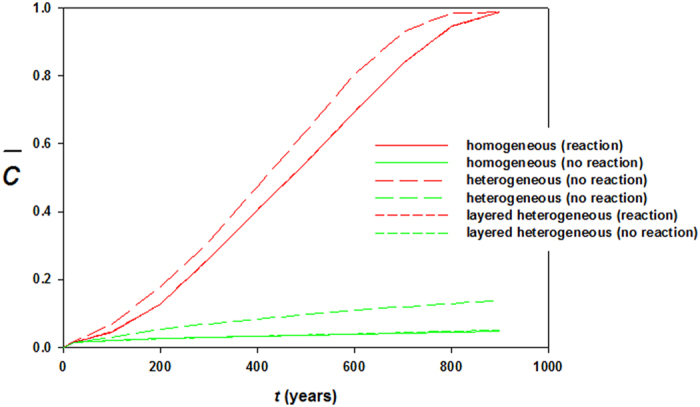
Average concentrations of dissolved CO_2_ in the reservoir for the case of *Ra* = 1000.

Figure 2 show time lapse of dissolved CO_2_ fronts from 20 years period to the time the reservoir domain becomes completely saturated. In the case of no reactions, because *Ra* is low, convection is diffusion dominated, resulting in very slow plume advancement. Even after 900 years CO_2_ propagation is still limited to upper 10% of the reservoir. However, reaction activities with calcite and dolomite make results completely different. By that time the entire porous formation becomes saturated with CO_2_. Whenever CO_2_ arrives at any particular domain after equilibrium reactions, CO_2_ stream in the form of 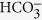 is dissolved from resident rocks, increasing local density of the brine. The increased density drives more instability which, in turn, causes plume boundaries to advance further downward. For this specific reservoir and thermophysical conditions in 900 years the pore volume is chock-full with dissolved CO_2_. The process adds significant feedback, however, in a negative sense because of early shutdown of both solubility and mineral trapping processes. Reactions constantly accelerate motion of the fronts. Because convective mixing shows diffusion dominance and the medium is homogeneous the cells do not form wormholes. Therefore, reaction fronts are planar throughout the process. No bifurcation occurs. Figure 3 exhibit results of same *Ra*, however for heterogeneous permeability formation. Dykstra-Parson coefficient of 0.55 was applied in generating the distributions shown in Fig. 4. Initially, results do not vary much from its homogeneous counterpart other than the pattern of plume evolution. As convection proceeds in addition to heterogeneity effects concurrent geochemical reactions make noticeable difference in dissolution process. Because of local permeability variations and nonlinear flow dynamics from the beginning of plume development hopf-bifuraction of the cells occur and CO_2_ stream added from carbonates help spread initially laterally and then finally dense phase sinks down. Thus, compared to no permeability contrasts, average concentration of CO_2_ in the aquifer differs significantly with time. ~100% saturation is reached 50 years earlier. On the other hand, <20% saturation only by reverse buoyant flow conveys clear message of reaction effects on CO_2_ convection (see Fig. 5). We have also tested results of layered anisotropic heterogeneity 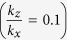. Mean permeability is too small to add any effect resulting in almost same results as homogeneous reservoir.

**Figure 6:**
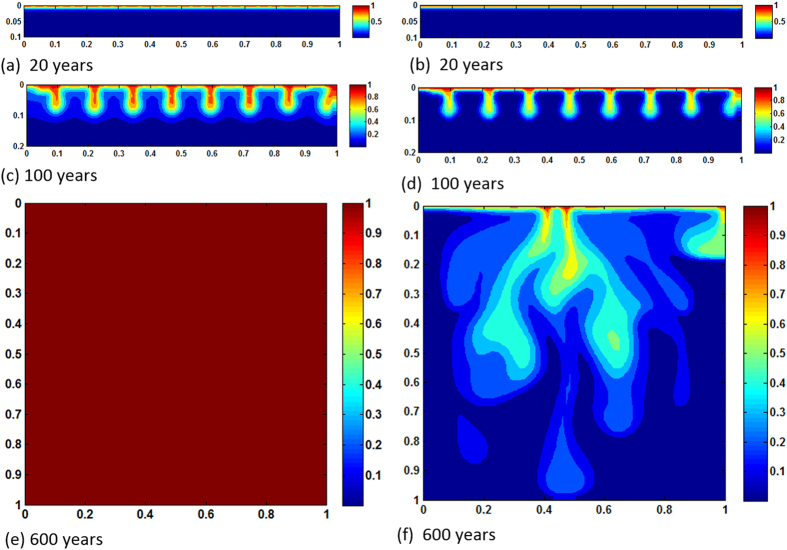
Concentration maps of *Ra* = 10,000, homogeneous case; (**a,c,e** and **b,d,f**) show results of with and without reactions, respectively.

**Figure 7:**
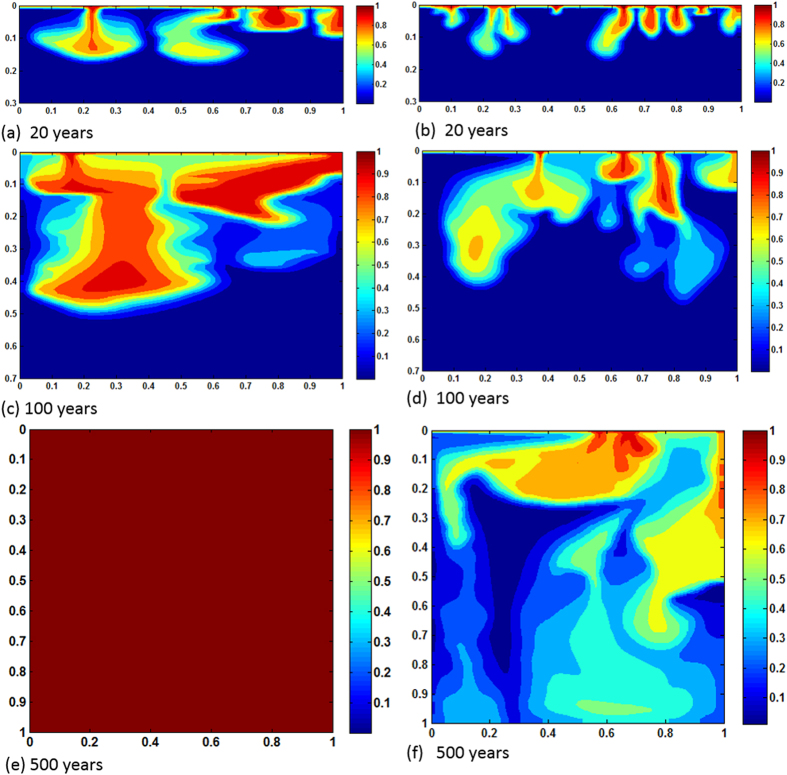
Concentration maps of *Ra* = 10,000, heterogeneous case; (**a,c,e** and **b,d,f**) show results of with and without reactions, respectively.

**Figure 8:**
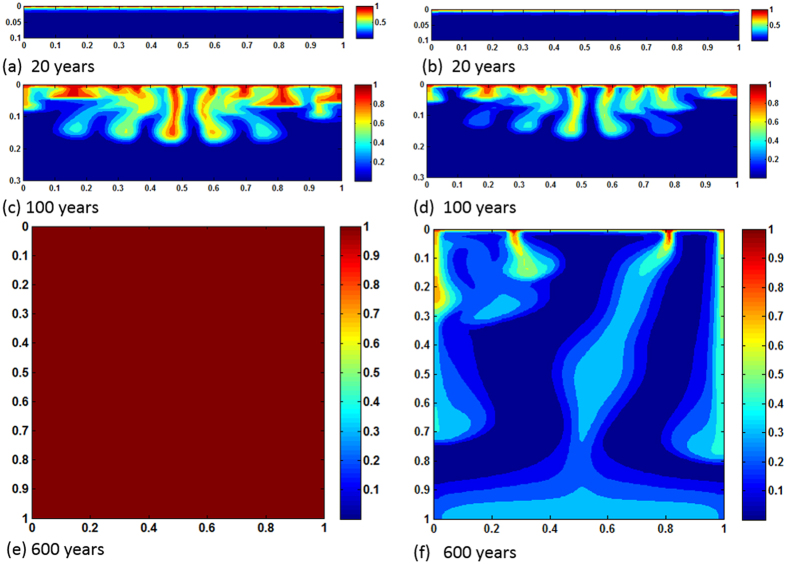
Concentration maps of *Ra* = 10,000, layered heterogeneous case; (**a,c,e** and **b,d,f**) show results of with and without reactions, respectively.

**Figure 9:**
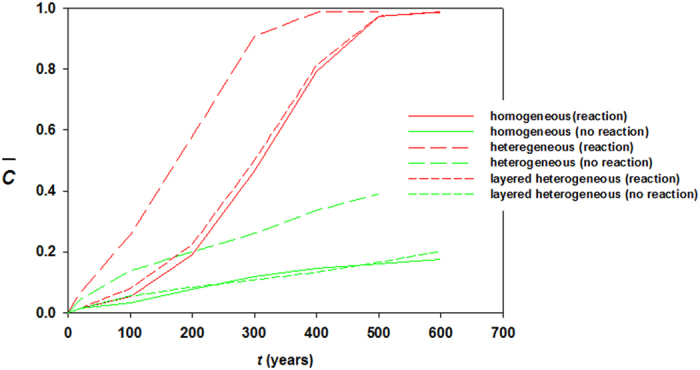
Average concentrations of dissolved CO_2_ in the reservoir for the case of *Ra* = 10,000.

Figures 6, 7, 8 plot concentration contours for the case of relatively high mean permeability 100 mD (*Ra* = 10,000). As expected, high permeability allows dissolving more CO_2_ and consequently reaction effects become more pronounced. Among three cases, the most intense enhancement is observed in heterogeneous reservoir where CO_2_ saturates completely in 500 years. *Ra* is high enough to initiate convection in early times and for heterogeneity the convection cells get more dynamicity because of availability of local high permeable favorable paths. Thus CO_2_ dissolution swells, transporting more CO_2_ saturated brine in contact with carbonates. The concurrent convection and reaction processes drive fast spreading of dense dissolved phase in the reservoir both laterally and downward. Figure 9 shows concentration profiles of *Ra* = 10,000. In this case layered heterogeneity adds little more reaction effect over the homogeneous distribution. For no reactions case maximum saturation obtained is ~40%. The early saturation due to geochemical effects has a negative effect on CCS in the sense that much injected CO_2_ may remain as completely undissolved. As a result, potential of leakage through high permeable zones or abandoned wells can be greater. To our best knowledge, no experimental results of convection enhancement due to geochemical reactions are reported. This strongly suggests a potential future research.

**Figure 10:**
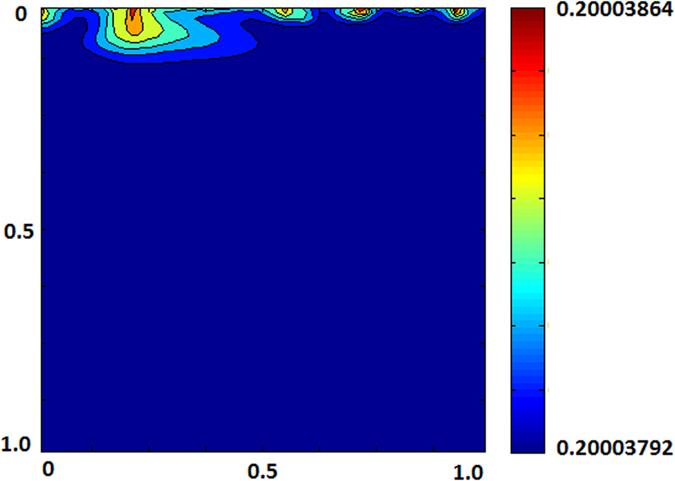
Change in porosity (*Ra* = 10,000, heterogeneous formation).

Figure 10 displays porosity changes, based on volume fraction deviations, for the case of *Ra* = 10,000 and heterogeneous reservoir. Though dissolution observed has significantly been boosted by the reactions, maximum porosity alteration until the reservoir becomes CO_2_ saturated is only 0.02%. This slight increase is almost negligible and cannot affect hydrodynamics of the system by any means. By using porosity-permeability relation^29^, 
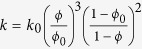
, respective permeability change is 0.06% which is also too small to distable fluid flow further.

## Methods (reservoir model and governing equations)

Following the model development in Islam *et al*.^30^ the continuity and transport equations read


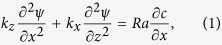



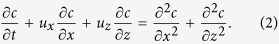


Here *c*, *t*, *u*, *k*, 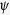 represent concentration, time, velocity, permeability, and the stream function. *x* and *z* are coordinates in lateral and vertical (positive downward) directions. *Ra* is the primary parameter that defines buoyancy driven flow. The bottom, lateral boundaries are impervious, and the top dense CO_2_-brine interface is perturbed with saturated dissolved CO_2_. Numerical solutions of Eqs. 1 and 2 are explained in details in Islam *et al*.^31^.

As dissolved CO_2_ comes into contact with rocks, it lowers the pH and triggers redistribution of carbonate aqueous species via the following reactions:


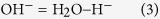



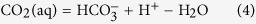



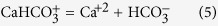



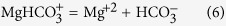



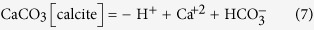



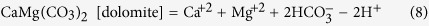


The chemical system involves eight reactive aqueous species (CO_2_(aq), H^+^, OH^−^, 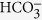, 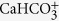, 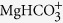, Ca^+2^, Mg^+2^), conservative species (Na^+^, Cl^−^) which are not involved in above reactions, and quartz (SiO_2_) matrix which remains inert. A sequential approach is used to first solve *c* from the conservative transport equation (Eq. 2), followed by coupling with equilibrium reactions Eqs 3, 4, 5, 6, 7, 8. The solution methods discussed in De Simoni *et al*.^32^ and Yang *et al*.^28^ are adopted for reactive transport. All required thermodynamics data (reaction constants, Debye parameter, species valence, etc.) are obtained from database file of PFLOTRAN (www.pflotran.org, accessed on Dec 20th, 2015) code. The reservoir and thermophysical data, and initial concentrations used are reported in Table 1.

## Conclusions

Convection from CO_2_-laden boundary is examined by coupling with reactive modeling of resident carbonate (calcite and dolomite) formations. Reactions involved were in locally equilibrium. We tested both homogeneous and heterogeneous cases with low and high mean permeability in term of *Ra* number. The sanity test delivered initial results of maximum equilibrium concentration of dissolved species. This showed dissolved CO_2_ stream from reactions can increase local density even more than one order of magnitude. Geochemistry based on reaction intensity can cause serious alteration of flow dynamics. Our numerical results provide clear message that upon affecting local concentrations density-driven convection is enhanced substantially. For instance, *Ra* = 10,000 and heterogeneous distributions show that reservoir reaches ~100% CO_2_ saturation in 500 years while only convective flow covers 40% upper area. Negligible porosity and permeability increase do not affect the hydrodynamics at all. The strong enhancement, in turn, adds negative impact on CCS in the sense that more free phase CO_2_ may exist in the storage formations, increasing the potential for leakage and prolonging the period for long-term monitoring.

## Additional Information

**How to cite this article**: Islam, A. *et al*. Reactive Transport Modeling of the Enhancement of Density-Driven CO_2_ Convective Mixing in Carbonate Aquifers and its Potential Implication on Geological Carbon Sequestration. *Sci. Rep*. **6**, 24768; doi: 10.1038/srep24768 (2016).

## Figures and Tables

**Table 1:** Data used in simulation.

Parameter	Value
Temperature (°C)	60
Pressure (bar)	100
Porosity	0.20
Permeability (mD)	10 or 100
Diffusivity (m^2^/s)	4.5 × 10^−10^
Viscosity (Pa.s)	0.001
Species	Initial concentration (mol/l)
CO_2_(aq)	0.0001
H^+^	1.148 × 10^−4^
OH^−^	0.8711 × 10^−8^
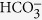	0.0073
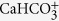	1.789 × 10^−4^
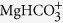	3.878 × 10^−5^
Ca^+2^	0.0022
Mg^+2^	4.89 × 10^−4^
